# The Impact of Menstrual Cycle Phase on Athletes’ Performance: A Narrative Review

**DOI:** 10.3390/ijerph18041667

**Published:** 2021-02-09

**Authors:** Mikaeli Anne Carmichael, Rebecca Louise Thomson, Lisa Jane Moran, Thomas Philip Wycherley

**Affiliations:** 1 Alliance for Research in Exercise, Nutrition and Activity (ARENA), Allied Health and Human Performance, University of South Australia, Adelaide, SA 5001, Australia; r.thomson@adelaide.edu.au (R.L.T.); tom.wycherley@unisa.edu.au (T.P.W.); 2 Adelaide Medical School and Robinson Research Institute, Faculty of Health and Medical Sciences, University of Adelaide, Adelaide, SA 5000, Australia; lisa.moran@monash.edu; 3 Monash Centre for Health Research and Implementation, School of Public Health and Preventative Medicine, Monash University, Clayton, VIC 3168, Australia

**Keywords:** sport, menstruation, female

## Abstract

The effect of the menstrual cycle on physical performance is being increasingly recognised as a key consideration for women’s sport and a critical field for further research. This narrative review explores the findings of studies investigating the effects of menstrual cycle phase on perceived and objectively measured performance in an athletic population. Studies examining perceived performance consistently report that female athletes identify their performance to be relatively worse during the early follicular and late luteal phases. Studies examining objective performance (using anaerobic, aerobic or strength-related tests) do not report clear, consistent effects of the impact of menstrual cycle phase on physical performance. Overall sport performance can be influenced by both perceived and physical factors. Hence, to optimise performance and management of eumenorrheic female athletes, there is a need for further research to quantify the impact of menstrual cycle phase on perceived and physical performance outcomes and to identify factors affecting variability in objective performance outcomes between studies.

## 1. Introduction

Elements of physiology unique to females, such as fluctuating female sex hormone concentrations throughout different phases of the menstrual cycle (MC), may be an important consideration for optimising the performance and maintaining the health of female athletes. Much of the research in the field of sports science has been conducted on males [1] and the findings of this research have been inappropriately applied to female athletes [2]. Studying the impact of MC phase on physical performance is one significant component needed to develop a female evidence base in sports science [2]. The development of this evidence base could enhance understanding of how the MC may impact athletes and inform how training, recovery and athlete monitoring programs are designed and delivered considering MC phase. 

### 1.1. Menstrual Cycle Physiology

The MC consists of a series of events that prepare the uterus for potential pregnancy. A MC that occurs regularly and lasts between 21 and 35 days is defined as eumenorrheic. A eumenorrheic MC is separated into two distinct main phases, follicular and luteal, which are established on the occurrence of menstruation, follicular maturation, ovulation and corpus luteum formation [3]. However, classifying the MC using just these two phases does not sufficiently distinguish the multiple hormonal milieus that occur within these two phases. Therefore, the MC is typically expressed in research using sub-phases, such as early follicular, late follicular, ovulatory, early luteal, mid luteal and late luteal [4].

**Figure 1:**
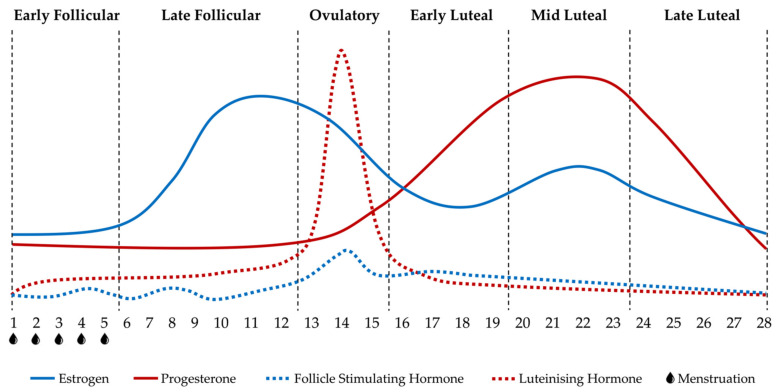
Hormonal events and phases in a eumenorrheic 28-day menstrual cycle. Adapted from McNulty et al. [5] and Farage et al. [6].

The fluctuations in female sex hormones, such as estrogen, progesterone, follicle stimulating hormone (FSH) and luteinising hormone (LH), presented in Figure 1, characterise the sub-phases of a eumenorrheic MC [3]. 

The early follicular phase begins with menstruation, which usually takes 4 to 6 days to complete; during this time, female sex hormone concentrations are all relatively low and stable. The follicular phase continues until ovulation occurs; during the late follicular phase, there is an increase in estrogen as the ovarian follicles, each containing an egg, mature. When estrogen rises to a critical point, there is increased secretion of gonadotropin releasing hormone, which then causes a rapid increase in LH. The LH surge, in the late follicular phase, triggers ovulation, in which a mature follicle ruptures releasing the egg into the uterus. The early luteal phase begins following ovulation; during this phase, the ruptured follicle becomes the corpus luteum and it secretes progesterone and a small amount of estrogen. The mid luteal phase contains the peak in progesterone and the second, smaller peak in estrogen, to prepare the endometrium for the implantation of a fertilised egg. The luteal phase will end with pregnancy if a fertilised egg is implanted. If the egg remains unfertilised, the corpus luteum will degrade, causing a decline in progesterone and estrogen during the late luteal phase as the cycle prepares to restart, with the uterine lining eventually detaching ready for menstruation to begin again [3,7,8]. The approximate timing of each MC phase is presented in Figure 1; however, the timing of ovulation, and therefore the various MC phases, can be highly variable [9]. This variability is the reason why measures such as urinary LH tests and serum estrogen and progesterone measurements are used to accurately identify MC phase [10,11].

The MC typically begins around 13 years of age [12] and will continue regularly until perimenopause around the age of 45 years [13] unless interrupted by pregnancy, hormonal contraceptive (HC) use or menstrual or ovulatory dysfunction. Approximately 67%- 91% of elite female athletes are eumenorrheic [14,15] and about half of eumenorrheic athletes do not use HC [16,17]. This suggests that a considerable proportion of female athletes may experience cyclic hormonal fluctuations.

### 1.2. Proposed Mechanisms behind Menstrual Cycle Based Changes in Performance

Physical performance has been postulated to change over the course of a MC due to various mechanisms, such as altered muscle activation, substrate metabolism, thermoregulation and body composition. Female sex hormone concentrations could be responsible for altered force production; this may affect muscle strength and power. Estrogen has a neuroexcitatory effect [18] and progesterone inhibits cortical excitability [18]; these neuroexcitatory and inhibitory effects result in estrogen and progesterone possessing a positive and negative relationship with force production, respectively [18,19,20,21]. It is hypothesised that greater strength and power outcomes would be produced when progesterone remains low during the follicular phase, especially when estrogen peaks during the late follicular phase, and lower strength outcomes would be produced in the luteal phase when progesterone is elevated. MC phase may also have a notable impact on rapid force production. Muscle activation, particularly the initial motor unit firing rate, is a main determinant of the rapid force production required to perform explosive movements [22]. In two studies, using females from the general population, the initial motor unit firing rate of the vastus medialis and vastus medialis oblique was significantly higher in the late luteal phase compared to the early follicular phase [23,24].

Another potential cause of altered strength is a MC-based change in bioavailable testosterone; this has been investigated in one study and there were no differences in strength or bioavailable testosterone between the early follicular and mid luteal phases [25]. Acute increases in testosterone can enhance physical performance via improved neural activation, muscle electrophysiological and contractile properties, and motor system function [26]. Other studies measuring basal salivary and plasma testosterone measured across multiple MC phases have revealed a peak in testosterone during the ovulatory phase [27,28] and post-exercise salivary and free testosterone were also increased in the ovulatory [27] and mid luteal phases [29]. It is unknown whether bioavailable testosterone increases during the ovulatory phase compared to other MC phases, when salivary and plasma testosterone concentration have been demonstrated to increase [27,28]. This mechanism should be researched further as it may result in fluctuations in strength over the MC.

Muscle and tendon stiffness have been investigated in a number of studies to establish whether MC phase is a risk factor for soft tissue injuries; some studies concluded that stiffness is affected by MC phase [30,31]. A relationship has been considered between stiffness of lower limb muscles and tendons and performance in tests that require reactive hops or jumps, such as short sprints or multiple jump tests [32,33], as greater stiffness provides for better storage and use of elastic energy [32]; MC phase could also alter performance via changes in tissue stiffness. It was suggested that the increased concentration of estrogen in certain phases of the MC may reduce stiffness by decreasing collagen synthesis and therefore collagen density in muscle and connective tissues [30]. Nevertheless, the results of studies investigating tissue stiffness over the MC are conflicting. In sedentary females, Achilles and patellar tendon, and medial gastrocnemius muscle stiffness was unchanged over the follicular, ovulatory and luteal phases [34]. In athletes, Achilles tendon strain did not differ between the early follicular and ovulatory phases [35]. It was concluded that any increase in estrogen during the MC is not great enough to result in a meaningful change in collagen synthesis and stiffness [35,36]. The effect of tissue stiffness may or may not vary throughout the MC and meaningfully impact performance.

The shift in thermoregulatory set point associated with elevated progesterone during the luteal phase may negatively or positively impact performance depending on the activity duration. Increased body temperature is known to improve performance in short duration activities requiring speed and power via better muscle contractility and force production [37]. It was proposed that the increased basal body temperature in the luteal phase could enhance short duration performance; however, it was found that sufficient active warm up protocol will negate the between-phase differences in basal body temperature and not affect performance [38]. In prolonged activities, the elevation in basal body temperature is suggested to impose greater thermoregulatory and cardiovascular strain, and potentially limit endurance performance, during the luteal phase [39,40,41]. Particularly, given internal body temperature before and after exercise is consistently elevated in the luteal phase, and cooling mechanisms indicated by sweat rate and skin temperature do not differ between phases [42].

Substrate availability and metabolism are also mechanisms proposed to vary in different phases of the MC and impact endurance performance. Estrogen supposedly increases the availability of free fatty acids for fuel during exercise and promotes lipid oxidation in skeletal muscle and progesterone counters the action of estrogen by limiting fat oxidation [43]. In a small sample of recreational athletes, when exercising at a high intensity (90% of lactate threshold), carbohydrate oxidation was lower and fat oxidation was greater during the mid to late luteal phase compared to the early follicular phase. Estrogen concentration was attributed to this change in metabolism as estrogen levels are typically lowest during the early follicular phase [44]. During submaximal exercise, carbohydrate and lipid oxidation remained similar between the mid luteal and late follicular phases [45,46]. Another study highlighted no change in free fatty acid availability and whole body peak lipid oxidation in three phases of the MC, despite significant changes in estrogen and progesterone [47]. The consumption of carbohydrates prior to exercise has also been found to negate the MC phase differences in relative carbohydrate and lipid oxidation during prolonged exercise [48]. The results from these studies do not provide a clear indication of how substrate metabolism during exercise may influence endurance performance but suggests it is possible that metabolism is affected by MC phase during exercise at higher intensities.

A possible mechanism behind altered performance may also be transient fluctuations in body composition throughout a MC. Increased body mass is associated with impaired aerobic endurance performance [49,50]. A decline in anaerobic performance is also associated with increased body mass, not resulting from increased lean body mass [51]. Like the evidence surrounding other proposed mechanisms, the effect of MC phase on body composition is not well understood. Various studies that measured body composition in female athletes in multiple MC phases determined that body mass [52,53,54,55], sum of skinfolds, [52,53], fat mass [52,55] and total body water [55] did not fluctuate over the MC. However, other studies demonstrated that body composition is affected. In athletes, body mass and total body water increased from the follicular to the luteal phase [56]. Similarly, the body mass [57] and total body water [58] increased from the follicular to the luteal phase in healthy, non-athletic females. This luteal increase in body mass could be caused by the decrease in insulin as progesterone increases, which drives appetite and food consumption [59,60], or by fluid retention as aldosterone is elevated during the luteal phase [61], and may influence athletes’ performance.

### 1.3. Menstrual Cycle Phase Monitoring in Elite Sport

The use of routine MC phase monitoring in elite sport is becoming increasingly widespread. Elite sporting organisations, including Chelsea Football Club and the United States’ Women’s National Soccer and Swimming Teams, have recently begun using a commercial smartphone app to track athletes’ MCs. The app is used by players to record when menstruation and various menstrual symptoms occur. Coaches and support staff can access the data entered into the app to identify if changes in an athlete’s performance or readiness occurred in different phases of the MC. Where cyclic changes in sleep, recovery and performance occurred, individual strategies were developed to address those changes, including modifications to an athlete’s sleep habits, training, diet or lifestyle factors based on guidance provided by the app [62,63,64].

The Brisbane Lions Australian Football Club has also been monitoring players’ MC since 2017 to ensure athletes do not develop relative energy deficiency syndrome [65]. A commercial app was used to track whether menstruation [66] and menstrual pain occurred [65]; this information enabled players and support staff to identify if individuals deviated from their typical cycles, and nutrition or training-based interventions could be implemented in these instances to ensure there was sufficient energy availability [65,66].

Given the increasing use of MC monitoring in applied sport science settings, it is important that there is high-quality research to inform the best-practice guidelines for whether and how training, nutrition or recovery programs should be modified based on MC phase.

## 2. Aim of Narrative Review

Two recent extensive systematic reviews and meta-analyses have examined the effect of MC phase on physical performance. Both reviews included studies from peer-reviewed journals, where physical performance was measured during two or more defined MC phases in eumenorrheic females, those that experience a MC regularly with a cycle length between 21 and 35 days [3], not using hormonal contraceptives (HCs) [5,67]. McNulty et al. [5] examined how exercise test performance was affected by MC phase in 78 studies, and found that there was a trivial reduction in exercise performance during the early follicular phase compared to other MC phases. The review conducted by Blagrove et al. [67] was performed on 21 studies that focused on the effect of MC phase on strength and power related performance and reported any fluctuations in strength and power that occurred throughout the MC were trivial to small, without a consistent effect of MC phase. These reviews have provided timely and important contributions to the expanding knowledge in the field. However, no general recommendations for the modification of training based on MC phases were able to be provided from either review due to the trivial effect sizes, high heterogeneity and low quality of the included studies [5,67], and it was suggested that more higher-quality research is required and any modifications to exercise programming and recovery should continue to be made on an individual basis [5].

These reviews included participants at various levels of conditioning from sedentary females to elite athletes [5,67], but it has been proposed the effect of MC phase on physical performance can differ between athletes and non-athletes [68]. The heterogenous population in addition to the large between study variance in performance outcomes investigated, test protocols, phases studied and methods of phase identification could have contributed to the trivial findings in these extensive reviews [5,67]. There are also no reviews, systematic or narrative, examining the effects of MC phase on perceived performance and the only applied physiological outcome investigated was maximal oxygen uptake [5]. Perceived performance is an important consideration as athletes’ beliefs towards a supposed ergogenic aid or factor, a means of enhancing performance [69], may result in an actual change in performance via a placebo effect [70].

The following narrative review aims to explore the impact of MC phase on perceived and objectively measured performance in athletic populations. It has been performed to complement the existing systematic reviews [5,67] by focusing solely on an athletic population, to reduce heterogeneity, and reporting for the first time on perceived performance and a range of applied physiological outcomes. Additionally, this review will identify current gaps within this evidence base and inform directions for future research.

## 3. Methods

A literature search was performed to identify relevant articles to include in this review; a description of the search strategy and screening process is provided in Appendix A. Studies were considered relevant if they recruited an athletic population: this includes individuals competing in any individual or team sport, or well-trained individuals that demonstrate a high level of conditioning or train at least four times per week. To be considered relevant, the articles must also have investigated athlete’s subjective assessments of their performance or objectively measured performance during exercise performance tests (maximal tests used to measure physical performance such as countermovement jump tests, time trials or maximal voluntary contraction tests) and applied physiological tests (maximal or submaximal tests that measure physiological variables associated with physical performance such as maximal oxygen uptake or fractional utilisation tests). The objective performance outcomes were categorised as strength performance if they measured force production or muscle activation via electromyography. Strength was delineated from anaerobic performance for the purpose of this review, depending on whether force or rate of force production was measured, for example when peak force (N) and peak power (W) were measured during a half squat exercise test [71], the force assessed strength performance and the power assessed anaerobic performance.

## 4. The Impact of Menstrual Cycle Phase on Perceived Performance

Five studies examining the effect of the MC on athletes’ perceived performance were included in this review. A summary of the included studies is presented in Table 1.

It is apparent that many athletes believe their performance fluctuates with MC phase; indeed, all included studies reported that MC phase impacted athletes’ perceived performance [72,73,74,75,76]. A large proportion, 50–71% and 49–65%, of participants reported feeling their performance in training and competition, respectively, is impaired in certain MC phases [72,74,76]. Perceived strength, speed and power ratings were also altered during certain MC phases [75]. Athletes most commonly perceived performance to be best in all phases of the MC except the early follicular and late luteal phases [76] and performance was perceived to be impaired in the early follicular and late luteal phases compared to the rest of the MC [72,75,76]. The perceived performance detriment in the early follicular and late luteal phases coincides with the occurrence of menstrual symptoms; a common reason attributed to this perceived performance decline was experiencing fatigue [72] or lethargy [74]—which are common menstrual symptoms experienced around menstruation in the general reproductive aged population [77]—menstrual pain and other menstrual symptoms [74]. Elite rugby players also cited feeling distracted by their menstrual pain or feeling concerned about flooding during the early follicular phase [74]. Distraction and worry about flooding was also reported by athletes from other sports and competitive levels [72].

Increased perceptions of fatigue in the early follicular and late luteal phases could be explained by the production of serotonin. Estrogen enhances serotonin transmission [78] and increases serotonin levels [79,80] via increasing production of tryptophan hydroxylase, which will increase the production of serotonin [81,82]. A study on 13 healthy females linked participants’ increased perception of tension-anxiety and fatigue in the late luteal phase with lower levels of serotonin, proposing that low estrogen concentration was responsible for the low serotonin levels [83]. Considering that estrogen remains relatively low in the early follicular phase, serotonin production and fatigue may remain increased in this phase, too. Females experiencing pre-menstrual syndrome and more severe menstrual symptoms, have also been found to have significantly lower serotonin levels in the mid and late luteal phases compared to age-matched regularly menstruating controls [84]. A cross-sectional study examining the effect of menstrual symptoms on performance reported that 44.6% of collegiate athletes believed menstrual symptoms impaired training or competition performance, and athletes that experienced symptoms of difficulty concentrating or feeling fatigued, and competed at an elite level were more likely to perceive impaired performance [85]. The studies included in this review did not specify if participants experienced pre-menstrual syndrome. Nonetheless it is interesting that fatigue and distraction were identified as risk factors for reduced performance [85], as these were some of the most common physical and psychological symptoms reported to have a negative impact on performance in the study on athletes [72].

## 5. The Impact of Menstrual Cycle Phase on Objectively Measured Performance

Thirty-five relevant studies were included in this review, a summary of the findings and characteristics of the included studies is provided in Table 2. Fifteen studies reported that at least one performance outcome was impacted by MC phase and 20 studies found no evidence that MC phase impacted performance. A summary of the findings and characteristics of the included studies is provided in Table 2. Overall, changes in objectively measured performance did not align with the changes in athletes’ perceived performance.

### 5.1. Effects of MC Phase on Anaerobic Performance

Sixteen of the included studies examined at least one anaerobic performance outcome; most of these studies (*n* = 13) determined that MC phase had no effect [38,52,53,54,71,95,96,100,106,107,108,110] and three studies demonstrated at least one anaerobic performance outcome fluctuated with MC phase [27,68,93]. Many outcomes derived from tests lasting 3 min or less demonstrated no effect from MC phase [38,52,53,68,71,95,96,100,106], which agrees with Eston and Burke’s proposal [112] that MC phase is unlikely to affect performances lasting 3 min or less in duration. Three studies assessing performance in short duration tests were concluded to be affected by MC phase [27,68,93]; 100 and 200 m sprint performance was better in the mid luteal phase [93], vertical jump height greater in the early follicular phase [68] and peak power during a repeated, short duration cycle ergometer sprints was significantly increased during the ovulatory phase [27]. The study finding that peak power was increased in the ovulatory phase, found participant’s motivation to train and compete was also greatest in this phase, potentially explaining this MC-based change in performance [27]. Hypothetically, this psychological affect could apply to performance in other tests; however, it should be researched further to determine if MC-based fluctuations in motivation affects a variety of performance outcomes. This ovulatory increase in peak power [27] may also be explained by estrogen’s role in increasing force production [18] as estrogen would likely still be elevated during the ovulatory phase following its late follicular peak or a potential elevation in testosterone during the ovulatory phase [27,28].

Performance in most sprinting and multiple jump tasks did not differ by MC phase [38,53,54,68,95,106,108,110]; as some authors purport tissue stiffness is unlikely to change over a MC [35,36], sprinting over short distances and reactive jump performance is unlikely to be affected by MC phase, or is affected by a different mechanism. Performance in other tasks requiring rapid force production was also largely unaffected by MC phase; explosive half squat [71] and countermovement [53,106], vertical [95] and squat jump test [106] outcomes did not vary by MC phase. One study demonstrated a significant decline in vertical jump height in the late luteal phase [68], which does not coincide with the greater initial motor unit firing rates observed during the late luteal phase [23,24] or any neuroexcitatory benefits of estrogen [18].

### 5.2. Effects of MC Phase on Muscular Strength

Compared to anaerobic and aerobic performance, muscular strength seems more likely to be affected by MC phase. Muscular strength, determined by tests measuring maximal voluntary contractions and force production, was reported in five studies as being affected by MC phase [21,68,91,101,103], whilst five studies reported no MC phase effects on strength [52,71,94,95,99] and one study reported a change in some strength outcomes and no change in other strength outcomes [89].

Two studies included in this review presented results consistent with the mechanism that strength is altered via estrogen and progesterone concentrations, and performance in strength tests was increased during the early [101] and late follicular phase [68]. Given that differences in strength would potentially be greatest between the late follicular and mid luteal phases, it is possible that no significant differences were observed in strength as strength outcomes were not compared between those phases [52,71,95]. However, multiple studies presented results that contradict this mechanism. Multiple studies found that there was no difference in strength between the late follicular and mid luteal phases [21,91,94,99], and strength outcomes were lower in the early follicular phase compared to the mid and late luteal phases [21], increased in the luteal phase [89], and significantly [103] and non-significantly [99] increased in the ovulatory phase.

The effect of MC phase on strength was examined in both dominant and non-dominant limbs in two studies [68,89]. There was a significant increase in dominant handgrip strength in the late follicular phase and there was significant, but unspecified, differences between phases in non-dominant handgrip strength [68]. The non-dominant hamstring to quadricep strength ratio was significantly decreased in the follicular phase compared to the luteal phase but there was no change in strength ratio of the dominant limb [89]. MC-based strength changes seem to differ based on limb dominance or muscle groups. The change in strength balance results from a relative increase in quadriceps and decrease in hamstring strength in the follicular phase, indicating that any effect of MC phase on strength may be augmented in different muscles or muscle groups. This is possible, as differential activation of the knee extensors has been observed in eumenorrheic females but not males, where the vastus medialis’ initial motor unit firing rate was significantly greater than the vastus medialis oblique’s during the ovulatory and mid luteal phases, but not in other MC phases [23].

### 5.3. Effects of MC Phase on Aerobic Performance

Six studies [53,88,91,92,104,109] demonstrated MC phase differences in aerobic exercise performance or applied physiological outcomes related to aerobic performance; two studies found some outcomes were affected by MC phase while others were unaffected [52,90], and the majority of studies (*n* = 14) concluded MC phase had no affect [46,52,54,86,87,93,96,97,98,105,107,108,110,111]. Continuous endurance performance measured with time to exhaustion tests [52,87,90], power output during maximal treadmill [86] and cycle [97,105] and rowing [111] tests and a 2000 m rowing time trial [93] was not affected by MC phase. The lack of effect on MC phase on various endurance performance tests [52,86,87,90,93,97,105,111] suggests that even if substrate metabolism was altered, it may not translate to impaired or improved performance.

Intermittent endurance performance appeared more likely to be influenced by MC phase than continuous endurance performance. Two studies concluded that athletes’ performance intermittent exercise tests were unaffected by MC phase [98,108,110], but improvements in the follicular phase have been observed; specifically, distance covered in a Yo-Yo Intermittent Endurance test increased in the early follicular phase [53], and during late follicular phase, fatiguability was reduced based on the lower fatigue index and the higher peak power in the final intervals of a repeated cycle sprint test [91]. Inconsistent with these results was the finding that repeated long duration sprint times were decreased in the late follicular, and early and mid luteal phases compared to the ovulatory and late luteal phases [104].

Performance in some intermittent endurance tests may have been greater in the follicular phases [53,91] due to the absence of increased thermoregulatory and cardiovascular strain during the luteal phase [39,40,41]. The finding that more runners produced their best marathon times during the luteal phase than the follicular phase [92] contradicts the hypothesis that increased strain during the luteal phase impairs performance, but this study did not consider confounders such as environmental conditions and the terrain or course undertaken, which may have impacted performance, and it only considered the best time recorded and it separated the MC using a simple two-phase model [92].

Mechanical efficiency may be influenced by MC phase, but the effect of MC on efficiency and whether any effect on efficiency results in impaired or improved performance is unknown [88,90]. During the mid luteal phase, participants from one study were found to be less economical [90], but another study determined participants were more economical in this phase [88]. Both studies utilised an incremental treadmill protocol; however, the length of the running and rest intervals differed from 5 min of running and rest in one protocol [88] and 3 min of running and 30 s of rest in the other [90]. It was suggested that impaired running economy in the mid luteal phase could be attributed to the shift towards greater thermoregulatory, cardiovascular and metabolic strain, but despite the reduced efficiency there was no corresponding reduction in time to exhaustion [90]. Performance was not recorded in the other study to confirm if the greater economy found in the mid luteal phase was enough to improve endurance performance, but the respiratory exchange ratio was recorded and did not vary between MC phases, suggesting that relative carbohydrate and lipid metabolism was not responsible for this change in running economy [88].

Maximal oxygen uptake is unlikely to alter aerobic performance in certain phases of the MC as it is frequently found to not be significantly different between MC phases [87,90,105,111], and just one study found a significant difference where absolute but not relative VO2max was greater in the early follicular phase compared to the mid luteal phase [52]. The findings of this review also indicate that endurance performance is unlikely to be impacted by fractional utilisation. The power output and oxygen uptake [105], and running speed [90] at the lactate threshold determined by the log-log and linear-regression breakpoint methods during cycle ergometer and treadmill testing, respectively, did not differ based on MC phase. Lactate accumulation also appears to be unaffected by the MC as the blood lactate concentration measured following submaximal [46] and maximal [54,96,98,105,107,111] exercise tests did not differ between MC phases [96,98,105,111].

## 6. Overall Impact of Menstrual Cycle Phase on Physical Performance

**Figure 2:**
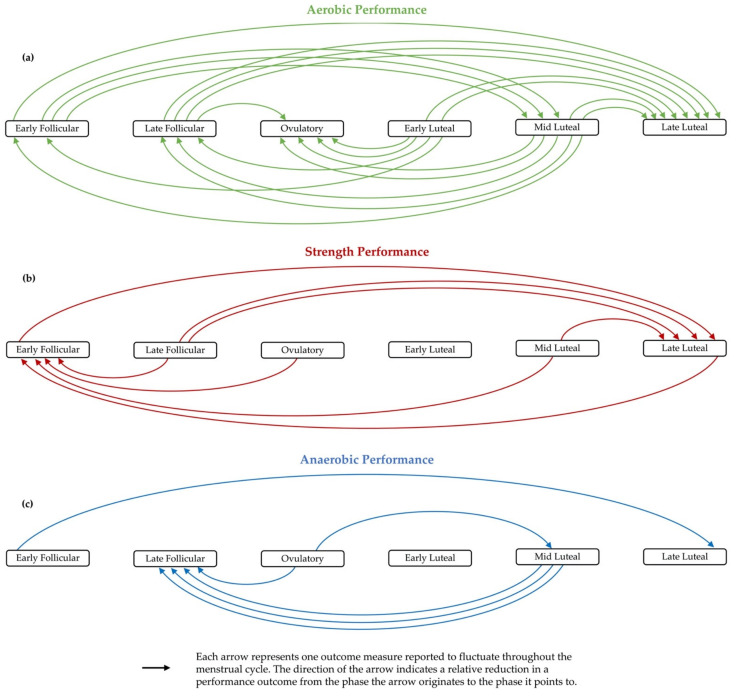
Summary of the changes in physical performance outcomes throughout the menstrual cycle observed in the included studies that reported that physical performance fluctuated across the menstrual cycle; (**a**) changes in aerobic performance outcomes; (**b**) changes in strength performance outcomes; (**c**) changes in anaerobic performance outcomes.

The trivial effect of MC phase on performance observed in previous systematic reviews [5,67] is understandable as the findings from this narrative review are mixed, with 20 of the 35 relevant studies concluding that MC did not produce significant improvements or impairments to physical performance, and 15 studies finding that at least one outcome was affected by MC phase. Seven of the twenty-four studies that measured performance in the early follicular and late luteal phases, when performance was perceived to be diminished [75,76], demonstrated a performance reduction in the early follicular [21,68,103,104] or late luteal phases [68,91,101,104,109] compared to other MC phases. Figure 2 demonstrates that more performance outcomes were relatively reduced in the late luteal phase than any other MC phase. This indicates that pre-menstrual syndrome, menstrual symptoms prior to menstruation or the declining female sex hormones may have a role in reducing performance. This also does not correspond with McNulty et al.’s [5] finding that overall performance was slightly reduced in the early follicular phase compared to other phases. However, the figure does not consider the magnitude of the between-phase performance reductions, only the frequency that performance outcomes are shown to be reduced, potentially explaining the conflicting conclusions between this narrative review and the systematic review.

Figure 2 also calls attention to the effect of MC phase on different performance variables and the inconsistent findings regarding the MC phase effects on performance. It appears that aerobic performance outcomes are likely to be enhanced but strength performance is diminished in the early follicular phase; similarly, endurance is diminished in the ovulatory phase, while strength and anaerobic performance improves in the same phase. There are also variations in the findings, for example, almost all outcomes measured in the late luteal phase were relatively reduced compared to the other MC phases, but one strength outcome was greater in the late luteal compared to early follicular phase. Based on this graphic, endurance performance is likely best early in the MC, and anaerobic and strength performance may be best in the ovulatory phase; strength and aerobic performance may be worst in the late luteal phase and anaerobic performance could be worst in the late follicular phase.

## 7. Limitations and Future Research

The studies included in this review provide important insights into the experiences of female athletes; however, there are some limitations in these studies. Three studies on perceived performance were cross-sectional; while this may have allowed for the recruitment of larger sample sizes, the responses rely on the participants’ recall over previous MCs. A recall bias has been found in many tools used to retrospectively assess menstrual pain [113,114]; it is possible there is also recall bias in these cross-sectional surveys and participants are over or underestimating the effect of the MC on performance. A similar bias may also have been present in the qualitative study as participants were required to recall the impact of previous MCs [74]. The longitudinal study removed this bias by recording perceptions during each phase of the MC. Instead of determining prevalence of perceived change throughout the MC, the study was able to measure the average perceived change in performance within a sample. However, the results may not be representative of whole menstruating collegiate athlete population due the study’s small sample size (*n* = 6) [75].

Future research could address these limitations by continuing to assess perceived performance and other subjective measures such as fatigue, soreness and motivation, using longitudinal or observational study designs with larger sample sizes to further the understanding of how and why athletes believe their performance fluctuates throughout the MC. Only one qualitative study in this area has been published; more studies adopting this methodology would be useful to better explore perceptions and experiences of menstruating athletes in depth, particularly about their concerns about performance at certain phases of the MC or exploring what barriers and facilitators exist to discussing the MC with coaching staff. Investigations of athletes’ readiness to train and compete throughout the MC are also required because readiness is a key factor that influences training adaptation and where an athlete is positioned on the overtraining continuum [102,115,116].

Sixteen studies in this review only investigated objective performance in two MC phases [46,52,53,87,88,89,92,93,98,100,102,105,107,108,111,115], ten of these studies also concluded that MC phase does not affect performance [46,87,98,100,102,105,107,108,111,115]. It is difficult to conclude whether a lack of an observed effect of MC phase was present as potential fluctuations during other phases were missed or MC phase had no bearing on the performance outcome. Going forward, research should follow the latest recommendations to assess outcomes in at least three phases, specifically the early follicular, ovulatory and mid luteal phases as these represent unique hormonal milieus [10] so any possible fluctuations are more likely to be identified.

The existence of low and high responders should also be investigated; Julian et al. [116] suggested that some athletes may be more or less susceptible to changes in performance as a result of MC phase. Given that some athletes report their performance to be affected by MC phase and others do not [72,76], and it is known that some females are more susceptible to experiencing severe menstrual symptoms than others [117], it is feasible there are individual differences in the extent to which MC phase affects performance. It would be useful to determine if low and high responders to these MC phase effects exist, and if they do, how these responders can be identified. Sub-group analyses conducted between athletes with and without pre-menstrual syndrome should also be performed to determine if pre-menstrual syndrome predisposes athletes to greater MC-based changes in performance.

The limitations of the research conducted on the effects of MC phase on objectively measured performance have been covered comprehensively in the recently published systematic reviews. The limitations reported by McNulty [5] and Blagrove [67] and their colleagues were similar and included the use of small sample sizes, absence of female sex hormone measurements to confirm MC phase, inconsistency in methods to determine MC phase and uncontrolled confounders. These limitations were also observed in this review. Only five of the 35 studies examining objectively measured performance included in this narrative review had more than 20 participants [68,89,92,93,95]. They also noted large variability in participant characteristics and training status [5,67]; however, the focus on an athletic and well-trained population means there are more homogenous samples in the studies included in this review. Some studies did include participants that competed at different levels or were athletes and non-athletes [27,68,95,111] but these studies analysed and interpreted the results of different groups separately.

In high performance sports, the difference between successful and unsuccessful performances can be minute, and a limitation of some studies included in this review is the reliance on statistical significance to determine if the effect of MC phase is meaningful to athletes. An example from a study included in this review was that a statistically significant difference in 100 m and 200 m freestyle swimming times was not observed between the early follicular and late luteal phase, but on average, participants swam 2.3 and 7.2 s faster in 100 and 200 m freestyle events, respectively, during the late luteal phase compared to the early follicular phase [100]. MC phase should be considered to have a considerable impact on swimming performance based on these findings, because 1.95 s separated the 1st and 8th placed female swimmers in the 100 m freestyle final during the 1992 Olympics [118]. The clinically significant, not just statistically significant, differences in performance need to be acknowledged in research in this space.

Approximately 13% (*n* = 91) of all participants included in this review were competing at international or national levels and eight studies investigated the MC effects on only field- or court-based team sport athletes. It is apparent that less research is conducted specifically on elite athletes and field-based team sport athletes, indicating future research could be conducted in these populations to address this gap in the literature. This is especially since training status may affect how the MC impacts performance [68,119], and field-based team sport athletes must possess a well-developed combination of power and endurance, whereas individual athletes typically specialise their training to primarily develop one performance variable [120].

Several observational studies have been conducted [53,100,102], in addition to two studies that did not meet the inclusion criteria provided in Appendix A, that retrospectively analysed the amount of low intensity and high intensity running that was performed during multiple training sessions [115] and games [116], respectively. Further studies using this design would be useful as various performance outcomes, from wearable devices or routine testing or monitoring, collected over multiple MCs during training sessions or competitive events such as games may be analysed.

Marathon performance was considered in one study that assessed what phase of the MC runners performed their fastest marathon using a cross-sectional survey [92]. Performance in prolonged, exhaustive exercise, such as triathlons, marathons or ultra-endurance events, should continue to be researched in experimental or observational studies. Long duration events may be more likely to observe altered performance as a result of increased lipid metabolism, which could lead to delayed glycogen depletion, when estrogen is elevated, or increased thermoregulatory and cardiovascular strain, when progesterone is elevated [39,40,41].

Examining the effects of HC use or menstrual dysfunction on athletes’ physical performance was beyond the scope of this review. Hormonal events typical of a eumenorrheic, ovulatory MC are not representative of the hormonal events in the cycle of HC users [121,122] or dysfunctional MCs, and an extensive review would be required to adequately discuss complexities such as the type of HC being used [121,122], the type of MC dysfunction [123,124,125] and related complications such as relative energy deficiency [126]. This current review also does not include discussion about athletes’ cognitive performance; however, we acknowledge the significant role of cognition in overall player performance in sports, particularly field-based team sports.

Other gaps in the literature include the efficacy of commonly used ergogenic aids throughout the MC, the effect of MC phase on agility performance and performance in different environmental conditions. Thus far, only the efficacy of caffeine supplementation during the early follicular, ovulatory phase has been studied in athletes [96,97], and only three studies tested performance in extreme environments [38,98,108]. The effect of beetroot juice on endurance performance in different phases of the MC should be assessed as female sex hormones have been attributed to changes circulating nitric oxide and nitric oxide synthesis [127,128] and the effect of these hormones on the exogenous nitrate to nitric oxide pathway and ergogenic effect in multiple MC phases is yet to be researched. Endurance performance, perceived performance or exertion in hot environments may be impaired during the luteal phase due to the shift in thermoregulatory set point and because cooling mechanisms do not appear to be upregulated during the luteal phase to counter the shift in thermoregulatory setpoint [42]. Further research on athletes’ performance in hot environments is necessary as many athletes are required to perform outdoors, in varying environmental conditions. Some studies have assessed lower body power, using tests such as countermovement jumps, which is correlated with agility in male athletes [129]. No studies included in this review have measured agility over the MC, despite agility being a significant performance determinant in field-based team sports [130,131]. It should be confirmed whether agility is unaffected by MC phase like other measures of lower body power, such as jumping and sprinting performance [38,53,68,95,106,110] or it is affected by MC phase, potentially as a result of factors other than power such as coordination being adversely affected by MC phase [132].

## 8. Conclusions

A substantial proportion of female athletes believe their performance is impacted by MC phase, but the research pertaining to objective measures of performance throughout the MC in eumenorrheic athletes does not provide a definitive indication of how performance may fluctuate throughout the MC. Many studies have concluded that performance does not vary between MC phases. In the studies that did observe a MC effect to performance, there were inconsistencies in findings but strength and aerobic performance were most commonly reported to be impaired during the late luteal phase, and anaerobic performance was most frequently reduced in the late follicular phase. With regards to perceived performance, the late luteal phase was also one of the phases athletes perceived their performance declined [75,76]. The research that finds the MC does have a mediating role in physical performance shows that MC phases affect strength, aerobic and anaerobic performance differently. If training is to be modified based on MC phase, the predominant performance variable being utilised and aims of training sessions must be carefully considered. In examining the literature, there are many questions related to the effect of MC phase on athletes that still need investigating, and in attempting to determine why performance may or may not fluctuate, it became evident there are also inconsistent findings on mechanistic outcomes, such as muscle and tendon stiffness or substrate metabolism, throughout the MC. Therefore, further research is needed to better the academic, athlete and applied sport science communities’ understanding of how the MC actually affects athletes’ performance and factors underpinning performance to inform decision-making and develop effective athlete management strategies to maximise performance and maintain health.

## Figures and Tables

**Table 1:** Details and findings of studies on the effect of the menstrual cycle on perceived performance in athletes.

1st Author (Year)	Study Design	Participants	Main Findings
Armour (2020) [72]	Cross-sectional	Athletes from various sports and competitive levels (*n* = 124), including non-HC users (*n* = 72)	50% of participants perceived training and 56.5% perceived competition was negatively affected in some MC phases. Some participants perceived some MC phases to have a positive effect on training (6.4%) and competition (<1%). Performance was most commonly reported to be affected in the EF and LL.
Ergin (2020) [73]	Cross-sectional	Elite volleyball athletes (*n* = 130)	84.6% of participants reported “sport-related menstrual problems” and 70.8% reported menstruation has affected their participation in training and competition in the EF.
Findlay (2020) [74]	Semi-structured interview	International rugby players (*n* = 15), including non-HC users (*n* = 11)	Majority of participants cited training (>66%) and competition (>50%) was negatively affected by the MC, particularly during the EF.
Jacobson (1999) [75]	Longitudinal	Collegiate athletes (*n* = 6)	Perceived strength and speed were, on average, significantly worse during the ML and LL. Perceived power was also, on average, significantly worse in both EF, ML and LL.
Solli (2020) [76]	Cross-sectional	Elite cross-country skiers and biathletes (*n* = 140), including non-HC users (*n* = 62)	51% and 71% of participants perceived training quality was positively and negatively affected by MC phase, respectively. 41% and 49% perceived competitive performance was positively and negatively affected by MCP, respectively. Performance was perceived to be worst during the EF.

*n* = sample size; MC = menstrual cycle; MCP = menstrual cycle phase; HC = hormonal contraceptive; EF = early follicular phase; LL = late luteal phase; ML = mid luteal phase.

**Table 2:** Details and findings of studies on the effect of the menstrual cycle on objectively measured performance in athletes.

1st Author (Year)	Study Design	Participants	Phases Studied	Main Findings
Burrows (2005) [86]	Randomised, counter-balanced based on MCP of first testing session	Highly trained 1500 m to marathon distance runners (*n* = 10)	EF, LF, EL and LL	⟷ Treadmill velocity at VO2max ⟷ Peak treadmill velocity
Cook (2018) [27]	Two group, longitudinal	National level (*n* = 6) and club/recreational (*n* = 16) figure shaping, soccer, netball or triathlon athletes	LF, OVU and ML	↑ Peak power (3 × 6 s cycle ergometer sprints) in OVU compared to LF and ML ↑ Improvement in peak power following post-activation potentiation stimulus in OVU compared to LF and ML
De Souza (1990) [87]	Two group, longitudinal	Well trained runners (*n* = 8)	EF and ML	⟷ Relative VO2max (incremental, maximal treadmill test) ⟷ TTE (incremental, maximal treadmill test)
Dokumaci (2019) [88]	Randomised, counter-balanced based on MCP of first testing session	Competitive athletes (*n* = 11)	LF and ML	↑ Running economy in ML compared to LF
Dos Santos (2017) [89]	Randomised, counter-balanced based on MCP of first testing session	Amateur female soccer athletes (*n* = 26)	FOL and LUT	↑ Non-dominant hamstring to quadriceps strength ratio in LUT compared to FOL ⟷ Dominant leg hamstring to quadriceps strength ratio
Goldsmith (2020) [90]	Longitudinal	Well trained runners (*n* = 10)	EF, LF and ML	↓ Running economy in ML compared to EF ⟷ Relative VO2max and TTE (incremental, maximal treadmill test) ⟷ Running speed at lactate threshold and 4 mmol/L blood lactate
Gordon (2013) [21]	Randomised, counter-balanced based on MCP of first testing session	Well trained females not using OC (*n* = 11)	EF, LF, ML and LL	↓ Knee extensor peak torque in EF compared to ML ↓ Knee flexor peak torque in EF compared to LL
Graja (2020) [91]	Randomised, cross-over based on MCP of first testing session	National-level handball athletes (*n* = 10)	LF, ML and LL	↓ Knee extensor MVC after repeated sprint protocol in LL compared to LF and ML ↓ Peak power (final 6 sprints of 20 × 5 s cycle ergometer sprints) in LL compared to LF ↓ % Decline in peak power (20 × 5 s cycle ergometer sprints) in LF compared LL
Greenhall (2020) [92]	Cross-sectional	Competitive, non-professional runners (*n* = 185)	FOL and LUT	↑ Percentage of runners (57.3%) recorded their best marathon time during LUT
Guo (2005) [93]	Longitudinal	Rowing or track and field athletes (*n* = 25)	LF and ML	↓ 500 m rowing ergometer, and 100 m and 200 m running times in ML compared to LF ⟷ 2000 m rowing ergometer time
Hertel (2006) [94]	Randomised, counter-balanced based on MCP of first testing session	Collegiate soccer or stunt cheerleading athletes (*n* = 14)	LF, OVU and ML	⟷ Knee flexor or extensor peak torque ⟷ Hamstring to quadriceps strength ratio
Julian (2017) [53]	Observational	High-level (National 2nd tier competition) soccer athletes (*n* = 9)	EF and ML	↓ Yo-Yo IET distance in ML compared to EF ⟷ 30 m running time ⟷ CMJ
Kishali (2004) [95]	Two group, longitudinal	Basketball, volleyball or judo athletes (*n* = 40)	EF, LF and LL	⟷ Vertical jump ⟷ Handgrip strength ⟷ 20 m running time
Lara (2019a) [96]	Double-blind, placebo-controlled, cross-over (randomly assigned MCP of first testing session)	Female triathletes (*n* = 13)	EF, OVU and ML	⟷ Peak power, mean power and fatigue index (15 s modified Wingate test) ⟷ Lactate accumulation ⟷ Magnitude of ergogenic effect of caffeine
Lara (2019b) [97]	Double-blind, placebo-controlled, cross-over, randomised (randomly assigned MCP of first testing session)	Female triathletes (*n* = 13)	EF, OVU and ML	⟷ Peak power (incremental, maximal cycle ergometer test) ⟷ Magnitude of ergogenic effect of caffeine
Lebrun (1995) [52]	Longitudinal	Trained runners, cyclists, triathletes, rowers, cross country skiers, or squash or ultimate frisbee athletes (*n* = 16)	EF and ML	↑ Absolute VO2max (incremental, maximal treadmill test) in EF compared to ML ⟷ Relative VO2max (incremental, maximal treadmill test) — Anaerobic Speed Test ⟷ TTE (90% VO2max treadmill test) ⟷ Knee flexor and extensor peak torque
Miskec (1997) [98]	Randomised, counter-balanced based on MCP and environmental condition of first testing session	Collegiate rugby players (*n* = 10)	EF and ML	⟷ Average power output (5 × 15 s cycle ergometer w/ 2 min active recovery) ⟷ Lactate accumulation
Otaka (2018) [99]	Counter-balanced, double-blind, placebo-controlled and crossover	Division 1 collegiate tennis athletes (*n* = 10)	EF, LF, OVU and ML	⟷ Dominant hip abductor and adductor peak torque ⟷ Dominant knee extensor peak torque
Quadagno (1991) [100]	Observational	Collegiate swimmers (*n* = 15)	EF and LL	⟷ 100 m and 200 m freestyle time
Rodrigues (2019) [101]	Randomised, counter-balanced based on MCP of first testing session	Well trained females (*n* = 12)	EF, LF and LL	↑ MVC (in 45° leg press) in EF compared to LL
Rogers (1996) [102]	Observational	Swimmers (*n* = 19)	FOL and LUT	⟷ Swimming times between FOL and LUT
Romero-Moraleda (2019) [71]	Randomised, counter-balanced based on MCP of first testing session	Female triathletes (*n* = 13)	EF, OVU and ML	⟷ Estimated half squat 1RM ⟷ Peak and mean force, velocity and power (20, 40, 60 and 80% of 1RM half squat)
Shahraki (2020) [103]	Cross-sectional	Collegiate athletes (*n* = 15)	EF, OVU and ML	↑ Dominant shoulder abduction, internal and external rotation strength in OVU compared to EF and ML
Shakhlina (2016) [104]	Longitudinal	High-level 800 m or 1500 m runners (*n* = 13)	EF, LF, OVU, (EL and ML), LL	↑ PWC170 in EL and ML compared to EF, LF, OVU and LL ↓ 4 × 400 m running time in EL, ML and LF compared to OVU and LL ↑ 4 × 400 m running time in EF compared to EL and ML
Smekal (2007) [105]	Randomised, counter-balanced based on MCP of first testing session	Healthy, sport participants (*n* = 19)	LF and LL	⟷ Peak power (incremental, maximal cycle ergometer test) ⟷ Absolute and relative VO2max (incremental, maximal cycle ergometer test) ⟷ Power output and VO2 at lactate and ventilatory thresholds ⟷ Lactate accumulation
Smirniotou (2004) [106]	Longitudinal	International level fencing athletes (*n* = 10)	EF, LF and LL	⟷ Squat jump ⟷ CMJ ⟷ Repeated jump
Somboonwong (2015) [38]	Longitudinal	National soccer athletes (*n* = 13)	EF and ML	⟷ 40 yard running time
Štefanovský (2016) [107]	Randomised, counter-balanced based on MCP of first testing session	Judo athletes (*n* = 8)	LF and ML	⟷ Peak power, mean power and fatigue index (Wingate test) ⟷ Lactate accumulation
Sunderland (2003) [108]	Randomised, counter-balanced based on MCP of first testing session	Well-trained game players (*n* = 7)	LF and ML	⟷ Loughborough Intermittent shuttle test ⟷ 15 m running time
Sutresna (2016) [109]	Longitudinal	Soccer and rowing athletes (*n* = 11)	EF (day 2), EF (day 5) and LL	↓ 1500 m running time in EF (day 5) compared to EF (day 2) and LL
Tasmektepligil (2010) [68]	Two group, longitudinal	Basketball, judo or football athletes (*n* = 30)	EF, LF and LL	↑ Dominant handgrip strength in LF compared to EF and LL Non-dominant handgrip strength fluctuates between phases (not specified) ↑ Vertical jump in EF compared to LL ⟷ 20 m running time
Tounsi (2018) [110]	Randomised, counter-balanced based on time of day of testing during each MCP	High-level soccer athletes (*n* = 11)	EF, LF and ML	⟷ Repeated shuttle-sprint ability test ⟷ Five-jump test ⟷ Yo-Yo IRT distance
Tsampoukos (2010) [54]	Randomised, counter-balanced based on MCP of first testing session	University soccer, hockey, track and field, basketball or rugby athletes (*n* = 14)	EF, OVU and ML	⟷ Peak power, mean power, peak speed, mean speed and fatigue index (2 × 30 s non-motorised treadmill sprints) ⟷ Lactate accumulation
Vaiksaar (2011a) [111]	Counter-balanced based on MCP of first testing session	National/international (*n* = 8) and recreational (*n* = 7) rowers	LF and ML	⟷ Absolute and relative VO2max (incremental, maximal rowing ergometer test) ⟷ Peak power (incremental, maximal rowing ergometer test) ⟷ Lactate accumulation
Vaiksaar (2011b) [46]	Counter-balanced study based on MCP of first testing session	National-level rowers (*n* = 11)	LF and ML	⟷ Lactate accumulation (70% VO2max rowing ergometer test)

*n* = sample size; MCP = menstrual cycle phase; OC = oral contraceptive; EF = early follicular phase; LF = late follicular phase; OVU = ovulatory phase; EL = early luteal phase; ML = mid luteal phase; LL = late luteal phase; FOL = follicular phase; LUT; luteal phase; VO2max = maximal oxygen uptake; TTE = time to exhaustion; VO2 = oxygen uptake; PWC170 = physical working capacity at heart rate of 170 beats per minute; IRT = intermittent recovery test; IET = intermittent endurance test; CMJ = countermovement jump; 1RM = one repetition maximum; MVC = maximal voluntary contraction; ↑ = increased; ↓ = decreased; ⟷ = no difference between MC phases.

**Figure A1:**
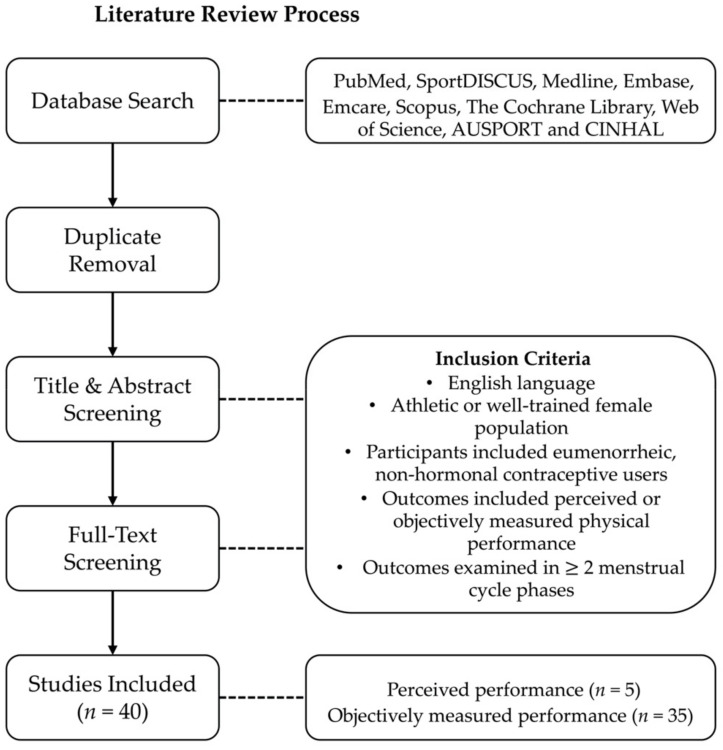
Summary of the search and screening methods implemented in this narrative review.

## Data Availability

No new data were created or analyzed in this study. Data sharing is not applicable to this article.

## Supplementary Materials

The following are available online at https://www.mdpi.com/1660-4601/18/4/1667/s1, Table S1: Complete Literature Search Strategy.

## Appendix A

A literature search was conducted in the following databases PubMed, SportDISCUS, Medline, Embase, Emcare, Scopus, The Cochrane Library, Web of Science, AUSPORT and CINHAL (final search 30 November 2020). The search strategy contained terms related to the MC and athletes. The full search strategy is contained in the Supplementary Materials (Table S1) and an overview of the search and screening process is provided in Figure A1.

Results from the database searches were imported in EndNote X9 (Clarivate Analytics), where duplicates were removed before screening occurred. One author conducted article screening to identify relevant articles, first based on whether the title and abstract was related to athletic performance over the menstrual cycle, followed by a full-text screening. Articles were included in this narrative review if they were published in English and if the population contained athletic or well-trained individuals. Participants were deemed to be athletic or well-trained if an author referred to participants as “athletes”, “highly trained” or “well-trained” or it was stated that the participants were participating in sport or training at least 4 days per week. One study did not refer to the participants as well-trained but was included in this review as the authors excluded participants based on their level of conditioning, ensuring only well-trained runners were included [90]. Participants must also have been described as eumenorrheic or having a “normal” MC, and not currently using any form of HC. No exclusion criteria were based on the participants’ competitive level or age.

Articles that fulfilled the criteria of including athletic or well-trained, eumenorrheic participants, must also have investigated participants’ perceived performance or objectively measured performance. Perceived performance were self-reported outcomes regarding how a participant believed they performed. Objective performance outcomes included were those derived from exercise performance tests (maximal tests used to measure physical performance such as countermovement jump tests, time trials or maximal voluntary contraction tests) and applied physiological tests (maximal or submaximal tests that measure physiological variables associated with physical performance such as maximal oxygen uptake or fractional utilisation tests).

Articles must also have compared physical performance in at least two phases of the MC. To standardise the results from the included articles, where possible, the phases were defined using the MC model established by McNulty et al. [5] consisting of the early follicular (days 1–5), late follicular (days 6–12), ovulatory (days 13–15), early luteal (days 16–19), mid luteal (days 20–23) and late luteal (days 24–28) phases.

After relevant articles were identified, one author extracted the following information, presented in Table 1 and Table 2: First author, year of publication, study design, participant information (sample size and description), phases investigated, and the findings of the study. A narrative review was then conducted to summarise the findings and identify the gaps within the literature.
