# Supplementary material for: The Impact of Menstrual Cycle Phase on Athletes’ Performance: A Narrative Review
Source: Int J Environ Res Public Health. 2021 Feb 9;18(4):1667. doi: 10.3390/ijerph18041667 (PMC7916245; doi:10.3390/ijerph18041667)
Supplement: Supplementary file 1 [file ijerph-18-01667-s001.pdf]

Complete Search Strategy

Title of the database searched<br>**PubMed**<br>Name of the database platform<br>**National Center for Biotechnology Information**<br>Complete search strategy<br>**("Athletes"[Mesh] OR "Sports"[Mesh]) OR ("athlete*"[Title/Abstract] OR "player*"[Title/Abstract] OR "competit*"[Title/Abstract] OR "paralympian*"[Title/Abstract] OR “train*”[Title/Abstract]) AND ("Menstrual Cycle"[Mesh] OR ("menstrual cycle*"[Title/Abstract] OR "uterine cycle*"[Title/Abstract] OR "ovarian cycle*"[Title/Abstract] OR "endometrial cycle*"[Title/Abstract] OR "reproductive cycle*"[Title/Abstract] OR "luteal phase"[Title/Abstract] OR "secretory phase"[Title/Abstract] OR "postovulatory phase"[Title/Abstract] OR "follicular phase"[Title/Abstract] OR "proliferative phase"[Title/Abstract] OR "preovulatory phase"[Title/Abstract] OR "menstruation"[Title/Abstract])) AND ("humans"[MeSH Terms] AND English[lang])**
Title of the database searched<br>**SPORTDiscus**<br>Name of the database platform<br>**EBSCOhost**<br>Complete search strategy<br>**((KW (athletes OR sports) OR SU (athletes OR sports) OR TI (athlete* OR player* OR competit* OR paralympian* OR train* OR sport*) OR AB (athlete* OR player* OR competit* OR paralympian* OR train* OR sport*)) AND (KW (menstrual cycle OR menstrual cycle phase*) OR SU (menstrual cycle) OR TI (“menstrual cycle*” OR “uterine cycle*” OR “ovarian cycle*” OR “endometrial cycle*” OR “reproductive cycle*” OR “luteal phase” OR “secretory phase” OR “postovulatory phase” OR “follicular phase” OR “proliferative phase” OR “preovulatory phase” OR menstruation) OR AB (“menstrual cycle*” OR “uterine cycle*” OR “ovarian cycle*” OR “endometrial cycle*” OR “reproductive cycle*” OR “luteal phase” OR “secretory phase” OR “postovulatory phase” OR “follicular phase” OR “proliferative phase” OR “preovulatory phase” OR menstruation))) AND LA english**
Title of the database searched<br>**MEDLINE**<br>Name of the database platform<br>**Ovid**<br>Complete search strategy<br>**(Menstrual Cycle.sh. or (menstrual cycle* or uterine cycle* or ovarian cycle* or endometrial cycle* or reproductive cycle* or luteal phase or secretory phase or postovulatory phase or follicular phase or proliferative phase or preovulatory phase or menstruation).ti. or (menstrual cycle* or uterine cycle* or ovarian cycle* or endometrial cycle* or reproductive cycle* or luteal phase or secretory phase or postovulatory phase or follicular phase or proliferative phase or preovulatory phase or menstruation).ab.) and ((Athletes or Sports).sh. or (athlete* or player* or competit* or paralympian* or train* or sport).ab. or (athlete* or player* or competit* or paralympian* or train* or sport).ti.) and limit 1 to (english language and humans)**
Title of the database searched<br>**Embase**<br>Name of the database platform<br>**Ovid**

Complete search strategy<br>**(Menstrual Cycle.sh. or (menstrual cycle* or uterine cycle* or ovarian cycle* or endometrial cycle* or reproductive cycle* or luteal phase or secretory phase or postovulatory phase or follicular phase or proliferative phase or preovulatory phase or menstruation).ti. or (menstrual cycle* or uterine cycle* or ovarian cycle* or endometrial cycle* or reproductive cycle* or luteal phase or secretory phase or postovulatory phase or follicular phase or proliferative phase or preovulatory phase or menstruation).ab.) and ((Athletes or Sports).sh. or (athlete* or player* or competit* or paralympian* or train* or sport).ab. or (athlete* or player* or competit* or paralympian* or train* or sport).ti.) and limit 1 to (english language and humans)**
Title of the database searched<br>**Emcare**<br>Name of the database platform<br>**Ovid**<br>Complete search strategy<br>**((Menstrual Cycle.sh. or (menstrual cycle* or uterine cycle* or ovarian cycle* or endometrial cycle* or reproductive cycle* or luteal phase or secretory phase or postovulatory phase or follicular phase or proliferative phase or preovulatory phase or menstruation).ti. or (menstrual cycle* or uterine cycle* or ovarian cycle* or endometrial cycle* or reproductive cycle* or luteal phase or secretory phase or postovulatory phase or follicular phase or proliferative phase or preovulatory phase or menstruation).ab.) and ((Athletes or Sports).sh. or (athlete* or player* or competit* or paralympian* or train* or sport).ab. or (athlete* or player* or competit* or paralympian* or train* or sport).ti.) and limit 1 to (english language and humans)**
Title of the database searched<br>**Scopus**<br>Name of the database platform<br>**Elsevier Science Publishers**<br>Complete search strategy<br>**TITLE-ABS-KEY ((("menstrual cycle*" OR "uterine cycle*" OR "ovarian cycle*" OR "endometrial cycle*" OR "reproductive cycle*" OR "luteal phase" OR "secretory phase" OR "postovulatory phase" OR "follicular phase" OR "proliferative phase" OR "preovulatory phase" OR "menstruation") AND ("athlete*" OR "player*" OR "competit*" OR "paralympian*" OR "train*" OR "sport"))) AND "human*" AND (LIMIT TO (LANGUAGE , "English"))**
Title of the database searched<br>**The Cochrane Library**<br>Name of the database platform<br>**John Wiley and Sons**<br>Complete search strategy<br>**((("menstrual cycle*" OR "uterine cycle*" OR "ovarian cycle*" OR "endometrial cycle*" OR "reproductive cycle*" OR "luteal phase" OR "secretory phase" OR "postovulatory phase" OR "follicular phase" OR "proliferative phase" OR "preovulatory phase" OR "menstruation") AND ("athlete*" OR "player*" OR "competit*" OR "paralympian*" OR "train*" OR "sport*"))) in Title Abstract Keyword - (Word variations have been searched)**
Title of the database searched<br>**Web of Science**<br>Name of the database platform

**Clarivate Analytics**<br>Complete search strategy<br>**(TS = ((("menstrual cycle*" OR "uterine cycle*" OR "ovarian cycle*" OR "endometrial cycle*" OR "reproductive cycle*" OR "luteal phase" OR "secretory phase" OR "postovulatory phase" OR "follicular phase" OR "proliferative phase" OR "preovulatory phase" OR "menstruation") AND ("athlete*" OR "player*" OR "competit*" OR "paralympian*" OR "train*" OR "sport*"))))**<br>***AND* LANGUAGE: (English)**
Title of the database searched<br>**AUSPORT**<br>Name of the database platform<br>**Informit**<br>Complete search strategy<br>**All Fields, Any Term ("menstrual cycle*" OR "uterine cycle*" OR "ovarian cycle*" OR "endometrial cycle*" OR "reproductive cycle*" OR "luteal phase" OR "secretory phase" OR "postovulatory phase" OR "follicular phase" OR "proliferative phase" OR "preovulatory phase" OR "menstruation") AND ("athlete*" OR "player*" OR "competit*" OR "paralympian*" OR "train*" OR "sport*")**
Title of the database searched<br>**CINAHL**<br>Name of the database platform<br>**EBSCOhost**<br>Complete search strategy<br>**((KW (athletes OR sports) OR SU (athletes OR sports) OR TI (athlete* OR player* OR competit* OR paralympian* OR train* OR sport*) OR AB (athlete* OR player* OR competit* OR paralympian* OR train* OR sport*)) AND (KW (menstrual cycle OR menstrual cycle phase*) OR SU (menstrual cycle) OR TI ("menstrual cycle*" OR "uterine cycle*" OR "ovarian cycle*" OR "endometrial cycle*" OR "reproductive cycle*" OR "luteal phase" OR "secretory phase" OR "postovulatory phase" OR "follicular phase" OR "proliferative phase" OR "preovulatory phase" OR menstruation) OR AB ("menstrual cycle*" OR "uterine cycle*" OR "ovarian cycle*" OR "endometrial cycle*" OR "reproductive cycle*" OR "luteal phase" OR "secretory phase" OR "postovulatory phase" OR "follicular phase" OR "proliferative phase" OR "preovulatory phase" OR menstruation))) AND LA english AND DH Human**
